# CRISPR/Cas9-Mediated Multiplex Genome Editing of the *BnWRKY11* and *BnWRKY70* Genes in *Brassica napus* L.

**DOI:** 10.3390/ijms19092716

**Published:** 2018-09-11

**Authors:** Qinfu Sun, Li Lin, Dongxiao Liu, Dewei Wu, Yujie Fang, Jian Wu, Youping Wang

**Affiliations:** Jiangsu Provincial Key Laboratory of Crop Genetics and Physiology, Yangzhou University, Yangzhou 225009, China; d150105@yzu.edu.cn (Q.S.); M160780@yzu.edu.cn (L.L.); DX120170119@yzu.edu.cn (D.L.); dewei@yzu.edu.cn (D.W.); yjfang@yzu.edu.cn (Y.F.)

**Keywords:** *Brassica napus*, CRISPR/Cas9, *WRKY*, Mutation pattern, *Sclerotinia sclerotiorum*

## Abstract

Targeted genome editing is a desirable means of basic science and crop improvement. The clustered, regularly interspaced, palindromic repeat (CRISPR)/Cas9 (CRISPR-associated 9) system is currently the simplest and most commonly used system in targeted genomic editing in plants. Single and multiplex genome editing in plants can be achieved under this system. In *Arabidopsis*, *AtWRKY11* and *AtWRKY70* genes were involved in JA- and SA-induced resistance to pathogens, in rapeseed (*Brassica napus* L.), *BnWRKY11* and *BnWRKY70* genes were found to be differently expressed after inoculated with the pathogenic fungus, *Sclerotinia sclerotiorum* (Lib.) de Bary. In this study, two Cas9/sgRNA constructs targeting two copies of *BnWRKY11* and four copies of *BnWRKY70* were designed to generate *BnWRKY11* and *BnWRKY70* mutants respectively. As a result, twenty-two *BnWRKY11* and eight *BnWRKY70* independent transformants (T_0_) were obtained, with the mutation ratios of 54.5% (12/22) and 50% (4/8) in *BnWRKY11* and *BnWRKY70* transformants respectively. Eight and two plants with two copies of mutated *BnWRKY11* and *BnWRKY70* were obtained respectively. In T_1_ generation of each plant examined, new mutations on target genes were detected with high efficiency. The vast majority of *BnWRKY70* mutants showed editing in three copies of *BnWRKY70* in examined T_1_ plants. *BnWRKY70* mutants exhibited enhanced resistance to *Sclerotinia*, while *BnWRKY11* mutants showed no significant difference in *Sclerotinia* resistance when compared to non-transgenic plants. In addition, plants that overexpressed *BnWRKY70* showed increased sensitivity when compared to non-transgenic plants. Altogether, our results demonstrated that *BnWRKY70* may function as a regulating factor to negatively control the *Sclerotinia* resistance and CRISPR/Cas9 system could be used to generate germplasm in *B. napus* with high resistance against *Sclerotinia*.

## 1. Introduction

The system of clustered, regularly interspaced, palindromic repeats (CRISPR)/Cas (CRISPR-associated) is the latest groundbreaking technology for genome editing and has become the dominant genome editing tool. The CRISPR/Cas system is used by bacteria and archaea as an RNA-guided defense system against invading viruses and plasmids [1,2]. CRISPR/Cas systems can be divided into three major types, namely, types I, II and III and the simplest and most commonly used system is CRISPR/Cas9, a type II system for *Streptococcus pyogenes* [3,4]. As an RNA-guided nuclease, Cas9 can be loaded into a single gRNA (sgRNA) engineered from two small RNAs (CRISPR RNA and trans-acting CRISPR RNA). The ribonucleoprotein complex formed by the sgRNA and Cas9 protein cleaves genomic DNA that is complementary to a 20 nucleotide stretch of the sgRNA as long as the 5′-NGG-3′ protospacer adjacent motif (PAM) is present in the complementary sequence [2].

Compared with zinc finger nucleases (ZFN) and transcription activator-like effector nucleases (TALEN), due to the ease of sgRNA manipulation, the CRISPR/Cas system presents advantages in terms of simplicity, accessibility, cost and versatility [5,6,7]. This system has been used successfully in many organisms, including animals [8,9,10], plants [11,12], fungi [13] and bacteria [14].

The CRISPR/Cas9 system can efficiently introduce several mutation types, including base substitutions [15,16], insertion mutations and deletion (indel) mutations [17,18] in the target site and deletions or inversions of a large chromatin fragment [19,20]. Unlike its predecessors, the CRISPR/Cas system can introduce a mutation in multiple sites simultaneously and can be used to edit several genes at the same time [21,22]. Therefore, this system is particularly useful for knockout of redundant genes or parallel pathways.

The genomes of model plants and cultivated crops including *Arabidopsis thaliana* [23,24], tobacco [16], tomato [18], rice [25,26], wheat [25,27], sorghum [21] and *B. oleracea* [28] have been successfully edited by CRIPSR/Cas9 system. This genetic modification technology does not require the persistent existence of foreign DNA and thus presents strong application prospects in crop breeding [7,11]. A few studies have presented targeted genome editing mediated by the CRISPR/Cas9 system in the important oil crop rapeseed. *ALCATRAZ* [29] *GA1-3*, *FRUITFULL*, *DA1*, *DA2* [30], *CLAVATA* [31] and *SPL3* [32], which are associated with plant or pod development; and *BnFAD2*, which is responsible for the desaturation of oleic acid to linoleic acid [33], were edited by the CRISPR/Cas9 system in *B*. *napus* by different groups. Most of the sgRNAs induced targeted editing, although there were a variety of editing efficiencies (5.3–100%) and the efficiency of multiple mutagenesis was significantly lower than that of single mutagenesis. However, to our knowledge, no attempt has been made to knockout pathogenesis-related genes by the CRISPR/Cas9 system to improve rapeseed resistance to pathogens. *S. sclerotiorum* is a nonspecific necrotrophic pathogen that causes sclerotinia stem rot in *B*. *napus*, resulting yield losses in oilseed Brassicas that vary between 5% and 100% [34]. Creating a new *Sclerotinia*-resistant variety has become the priority goal of crop breeders [35].

WRKY transcription factors (TFs), defined by their DNA-binding domain, namely, the WRKY domain, have been identified in different plants [36,37] and are widely involved in defense to diverse plant stress conditions, especially in plant immune responses [38,39,40,41]. In *Arabidopsis*, many WRKY transcription factors have been reported to be associated with disease resistance, including *WRKY8* [42], *WRKY11* [43], *WRKY33* [44,45], *WRKY38* and *WRKY62* [46], *WRKY46* [47], *WRKY53* and *WRKY70* [48]. Studies have shown that overexpression or loss function of *WRKY11* or *WRKY70* affects SA and JA-induced disease resistance response to pathogens in *Arabidopsis* [43,49,50,51]. Previous reports suggest that some *BnWRKY* genes might be involved in the response to pathogens in *B*. *napus* as well [52,53,54,55].

In the present study, we explored the patterns of targeted mutagenesis of the *B*. *napus* genome mediated by the CRISPR/Cas9 system. CRISPR/Cas9 vectors with multiple sgRNA expression cassettes were constructed to target the *BnWRKY11* and *BnWRKY70* genes of *B*. *napus* and *Agrobacterium*-mediated genetic transformation was used to generate transgenic plants. The mutations of targeted sites were then investigated by amplifying and sequencing in the T_0_ and T_1_ generations. The mutation pattern was analyzed as well. *S*. *sclerotiorum* resistances of the *BnWRKY70* knockout and overexpression plants were assessed by detached leaf inoculation and it turned out that loss function of *BnWRKY70* enhanced, while overexpression of *BnWRKY70* reduced resistance to *S*. *sclerotiorum*. Our findings suggested that the CRISPR/Cas9 system can be used to generate multiple homologs mutated plants in *B*. *napus*. With the high editing efficiency of this system in T_1_ plants, homozygous mutants can be generated in limited generations. Therefore, the CRISPR/Cas9 system could be an effective method for theoretical research and could improve rapeseed resistance to pathogens.

## 2. Results

### 2.1. Sequence Identification and Expression Analysis of BnWRKY11 and BnWRKY70 Genes in B. napus

**Figure 1:**
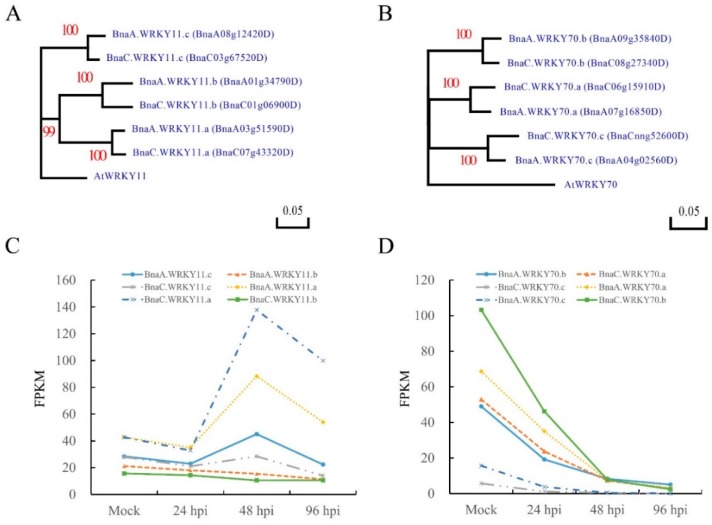
Phylogenetic tree of *WRKY11* and *WRKY70* and the expression level of *BnWRKY11* and *BnWRKY70* in response to *S*. *sclerotiorum* inoculation. (**A**) Phylogenetic tree of *BnWRKY11* and the homologs from *Arabidopsis*; (**B**) Phylogenetic tree of *BnWRKY70* and the homologs from *Arabidopsis*; (**C**,**D**) The expression level of *BnWRKY11* and *BnWRKY70* in response to *S*. *sclerotiorum* inoculation [51]. The tree was generated using the DNAMAN program by maximum likelihood (ML) methods. Bootstrap values are displayed with red numbers. hpi, hours post-inoculation.

Wu et al. [56] analyzed the transcriptome of *B*. *napus* lines to investigate the defense responses to *S*. *sclerotiorum* using in-depth RNA sequencing (RNA-seq), results showed that *BnWRKY11* and *BnWRKY70* genes differentially expressed in resistant *B*. *napus* lines J964 after inoculated by *S*. *sclerotiorum*. Both *AtWRKY11* and *AtWRKY70* genes have one copy in *Arabidopsis* [57]. Depending on the *AtWRKY11* and *AtWRKY70* gene sequence, we found the reference genome of Darmor-bzh [58] comprised six homoeologs of *BnWRKY11* and *BnWRKY70* genes respectively by BlastP (E-value ≤ 1 × 10^−5^, identity ≥ 50% and coverage ≥ 50%) (Figure 1A,B). Depending on the naming conventions of Østergaard et al. [59], the copies of *BnWRKY11* and *BnWRKY70* were named *BnaA.WRKY11.a*, *BnaA.WRKY11.b*, *BnaA.WRKY11.c*, *BnaC.WRKY11.a*, *BnaC.WRKY11.b*, *BnaC.WRKY11.c* (Figure 1A), and *BnaA.WRKY70.a*, *BnaA.WRKY70.b*, *BnaA.WRKY70.c*, *BnaC.WRKY70.a*, *BnaC.WRKY70.b*, *BnaC.WRKY70.c* (Figure 1B) respectively. According to the transcriptomics sequencing data published by Wu et al. [56], we found that three of the six copies (*BnaA.WRKY11.a*, *BnaC.WRKY11.a* and *BnaA.WRKY11.c*) were significantly up-regulated at 48 h post-inoculation (hpi) (Figure 1C), *BnaA.WRKY11.a* and *BnaC.WRKY11.a* not only showed the greatest expression change after inoculation but also had highest expression level before inoculation than those of the other four copies (Figure 1C). The expression of six *BnWRKY70* homologue genes were significantly down-regulated after inoculated by *S*. *sclerotiorum* and the expression level were getting lower and lower over inoculation time (Figure 1D). The expression level of *BnaA.WRKY70.c* and *BnaC.WRKY70.c* were significantly lower than that of other four copies. Among the *BnWRKY11* and *BnWRKY70* genes, *BnaC.WRKY11.a* and *BnaC.WRKY70.b* had the highest expression levels before inoculation with *S*. *sclerotiorum* and also most significantly induced (*BnaC.WRKY11.a*) or suppressed (*BnaC.WRKY70.b*) after inoculation. Because of the difficulty in simultaneously targeted editing to up to six copies, the copies of *BnWRKY11* and *BnWRKY70* that have high initial expression level and most dramatically induced or suppressed after inoculation with *S*. *sclerotiorum* were chosen as candidate genes to knockout by CRISPR/Cas9 system. For *BnWRKY11*, *BnaA.WRKY11.a* and *BnaC.WRKY11.a* were chosen and for *BnWRKY70*, *BnaA.WRKY70.a*, *BnaA.WRKY70.b*, *BnaC.WRKY70.a* and *BnaC.WRKY70.b* were chosen.

### 2.2. CRISPR/Cas9 Binary Vector Construction, Rapeseed Transformation and Screening of Positive Transformants

**Figure 2:**
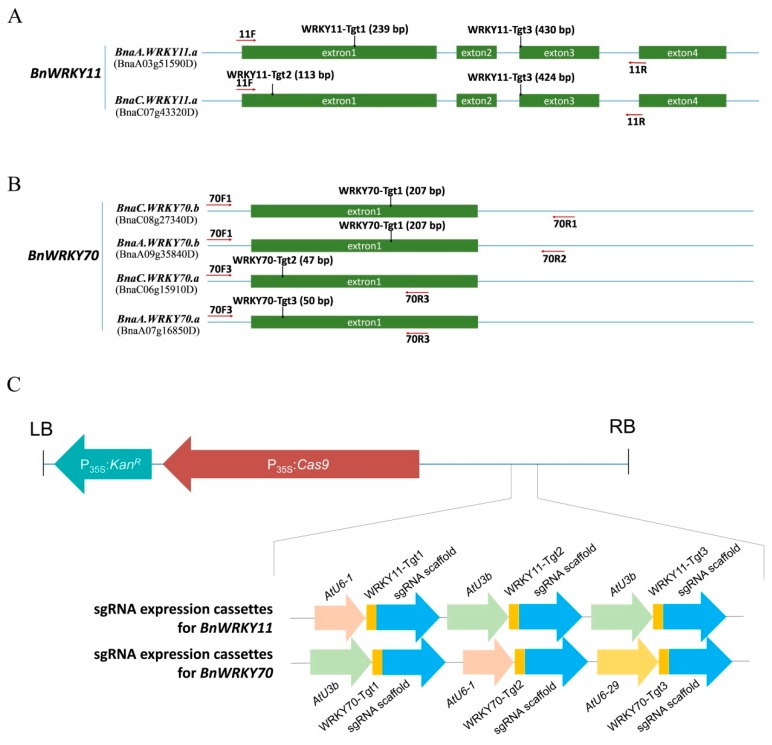
Position of target sites and primers on *BnWRKY11* and *BnWRKY70* and physical maps of the T-DNA regions of Cas9/sgRNA constructs. (**A**,**B**) the target sites for *BnWRKY11* and *BnWRKY70* respectively and the primers for the amplification were shown as well. Tgt1-Tgt3 means the chosen target sites, the locations of target sites are marked with black arrows; primers are shown in red arrows. (**C**) Physical maps of the T-DNA regions of Cas9/sgRNA constructs. LB/RB, left/right border of T-DNA; P_35S_:*Cas9*, *Cas9* gene which driven by CMV35S promoter; P_35S_:*Kan^R^*, *NTP* gene which driven by CMV35S promoter. AtU3/AtU6, *Arabidopsis* U3/U6 promoter.

We targeted *BnWRKY11* and *BnWRKY70* genes in *B*. *napus* to test the CRISPR/Cas9 system for genome editing (Figure 2). For *BnWRKY11*, we designed two sgRNAs targeting *BnaA.WRKY11.a* and *BnaC.WRKY11.a*. WRKY11-Tgt1 (Target1) and WRKY11-Tgt3 targeted the first and third exons of *BnaA.WRKY11.a* and WRKY11-Tgt2 and WRKY11-Tgt3 targeted to the first and third exons of *BnaC.WRKY11.a*, respectively (Figure 2A). All three WRKY70-Tgt targeted the first exon of the WRKY70 genes and WRKY70-Tgt1 targeted *BnaC.WRKY70.b* and *BnaA.WRKY70.b*, while WRKY70-Tgt2 targeted *BnaC.WRKY70.a* and WRKY70-Tgt3 targeted *BnaA.WRKY70.a* (Figure 2B). CRISPR/Cas9 constructs that targeted to *BnWRKY11* and *BnWRKY70* with three sgRNA expression cassettes were generated (Figure 2C). The binary expression vector pLYCRISPR/Cas9P_35S_-N containing a *neomycin phosphotransferase* gene was used for genetic transformation. With G418 as the selection agent and as confirmed by polymerase chain reaction (PCR) (primers: *Cas9*-F: GAAGTACCCCACTATCTACCAC, *Cas9*-R: ATGAAGAGCTTGTCCACGTC), we obtained 30 transgenic plants with 22 *BnWRKY11* transformants (CRI-W11) and 8 *BnWRKY70* transformants (CRI-W70).

### 2.3. Confirmation of Cas9-Induced Mutagenesis in Transgenic Plants of B. napus

To detect mutagenesis at the targeted site, we cut and mixed several leaves from the transgenic plants for DNA extraction. Using locus-specific primers (Table S2), we amplified and sequenced the flanking sequences in given target sites. As expected, a double-peak phenomenon occurred 3–4 bp upstream of PAM in the sequence chromatograms of amplicons (Figure S1).

The Sanger chromatograms of the PCR products of the targeted DNA were analyzed by the online tool TIDE (Tracking of Indels by Decomposition, https://tide.deskgen.com) [60] to evaluate the existence of editing events and mutation efficiency with *p*-value < 0.001 (Tables S4 and S5). Among the twenty-two T_0_ transgenic lines of CRI-W11, genomes of twelve and ten plants were edited at WRKY11-Tgt2 and WRKY11-Tgt3 sites in *BnaC.WRKY11.a* respectively, while eight plants among them showed mutated in both copies of *BnWRKY11* (Table 1 and Table S4). No editing events were detected at WRKY11-Tgt1 site. Among the eight CRI-W70 transgenic plants, three independent mutagenesis were induced by WRKY70-Tgt2 and WRKY70-Tgt3 in the *BnaA.WRKY70.b* and *BnaA.WRKY70.a* loci, respectively (Table 1, Figure S1). This represents that mutation frequencies were 54.5% at WRKY11-Tgt2 (*BnaC.WRKY11.a*), 31.8% at WRKY11-Tgt3 (*BnaA.WRKY11.a*) and 40.9% at WRKY11-Tgt3 (*BnaC.WRKY11.a*) in T_0_ plants of CRI-W11. 37.5% plants were mutated by WRKY70-Tgt2 and WRKY70-Tgt3 at *BnaA.WRKY70.b* and *BnaA.WRKY70.a* respectively (Table 1). Two of the CRI-W70 plants showed mutated in both *BnaA.WRKY70.b* and *BnaA.WRKY70.a* (Table 1 and Table S5).

**Figure 3:**
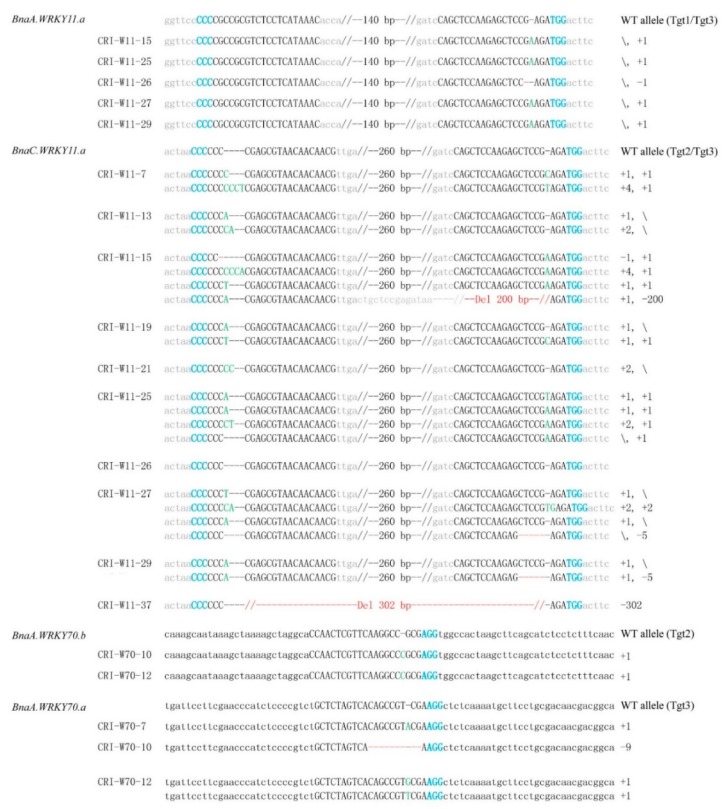
Multiplex mutagenesis of *B*. *napus* genome in T_0_ generation. The protospace adjacent motif (PAM) is shown in bold blue letters; red dashes mark the deletions; the inserted nucleotide is marked by a green letter. The numbers on the right show the type of mutation and how many nucleotides are involved, with “−” and “+” indicating deletion or insertion of the given number of nucleotides, respectively. Tgt1-Tgt3 means the target sequence used to generate sgRNA expression cassette.

To identify the mutation type, we cloned the mutated amplification products and then randomly sequenced six clones. Depending on the mutation efficiencies assessed by TIDE, some samples with low mutation efficiency were not analyzed by sequencing. The results showed that one or more editing events occurred at the target sites of these transgenic lines (Figure 3). Four alleles were detected in the transgenic plants CRI-W11-15, CRI-W11-25 and CRI-W11-27 and 3 different alleles were detected in CRI-W11-7, CRI-W11-13, CRI-W11-19 and CRI-W11-29 including the WT allele, indicating that the plants were chimeric. In addition, a deletion of 302 bp in *BnaC.WRKY11.a* of the CRI-W11-37 plant was detected (Figure 3). Notably, the potential double-strand breaks at WRKY11-Tgt2 and WRKY11-Tgt3 sites in *BnaC.WRKY11.a* were 302 bp distant and therefore, targeted genomic deletion was achieved between Cas9 cut sites. The sequencing results showed that three types of *BnaA.WRKY70.a* alleles existed in CRI-W70-12, including a WT allele (Figure 3). Among the 6 targets, 4 of them (WRKY11-Tgt2, WRKY11-Tgt3, WRKY70-Tgt1 and WRKY70-Tgt3) induced mutations with different editing efficiencies, whereas the other 2 targets did not. These results suggest that the CRISPR/Cas9 system can be used to edit more than one gene simultaneously in *B*. *napus* and that targeted genomic deletion can be achieved by multiplex editing with a relatively low efficiency.

### 2.4. Variety and Frequency of Mutations

**Figure 4:**
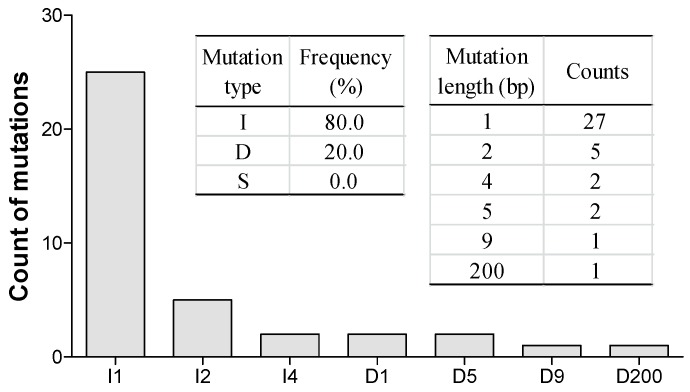
Mutation types and frequency in transgenic plants. Mutation types and frequency from combined data of four different targets at T_0_ generation. Left insert, occurrence of insertion (I), deletion (D) and substitution (S) mutation types. Right insert, counts of different mutation length. In x-axis: I^#^, ^#^ of bp inserted at target site; D^#^, ^#^ of bp deleted from target site.

In the current study on *B*. *napus*, the mutation types and frequencies were surveyed in the T_0_ generation of transgenic plants (Figure 4). Using the limited number of editing events in T_0_ plants, we summarized the mutation types induced by the sgRNA we used in this research. Results showed that, among the detected mutations, 80% (32/40) were insertions and the remaining 20% (8/40) were deletions; no substitutions were found. Most of the insertions were 1 bp (25/40). Six deletions that ranged from 1–200 bp were detected. 27 of 40 all mutations we detected in T_0_ plants changed by only 1 bp. All identified mutations occurred between bases 3 and 4 upstream of the PAM of the given target site.

### 2.5. Mutagenesis in T_1_ Plants

The alleles of the targeted genes of the T_1_ plants were examined by sequence analysis of the T_1_ plants CRI-W11-6, CRI-W11-10, CRI-W70-6, CRI-W70-7, CRI-W70-9 and CRI-W70-10. For the T_0_ generation of the transgenic plants we chose, genome editing events were not detected at all targets in CRI-W11-6, CRI-W11-10, CRI-W70-6 and CRI-W70-9 plants (data not shown), while CRI-W70-7 showed heterozygous mutations at WRKY70-Tgt3 (targeting *BnaA.WRKY70.a*) and CRI-W70-10 showed heterozygous mutations at both WRKY70-Tgt2 (targeting *BnaA.WRKY70.b*) and WRKY70-Tgt3 (targeting *BnaA.WRKY70.a*) (Figure 3).

Results of mutation detection showed that many new editing events occurred in T_1_ plants (Table S3). In T_1_ plants of the CRI-W11-6 and CRI-W11-10 lines, we detected mutation efficiencies of WRKY11-Tgt2 (targeted to *BnaC.WRKY11.a*) and WRKY11-Tgt3 (targeted to *BnaA.WRKY11.a* and *BnaC.WRKY11.a*) reaching 100% (Table 2). All 4 lines of CRI-W70 showed a high proportion of mutagenesis in *BnaA.WRKY70.b* (8/10–10/10), *BnaC.WRKY70.a* (8/10–10/10) and *BnaA.WRKY70.a* (7/10–10/10) (Table 2). However, no mutagenesis was mediated by WRKY11-Tgt1 (targeting *BnaA.WRKY11.a*) or WRKY70-Tgt3 in any of the T_1_ plants. TA cloning and sequencing of the targeted sequences were performed in T_1_ plants as well. The results showed some T_1_ plants of the CRI-W11 and CRI-W70 lines were chimeras (Table S3). These results indicated that compare to T_0_ plant, additional mutations happened in T_1_ plants.

The existence of the CRISPR/Cas9 component in T_1_ plants was also examined. Ten T_1_ transgenic plants were randomly selected and DNA of the leaves was extracted and amplified. Among the T_1_ plants examined (Table 2), segregation of the CRISPR/Cas9 components was detected in the CRI-W70-6, CRI-W70-7 and CRI-W70-10 lines, while the CRISPR/Cas9 component was detected in all of the CRI-W11-6, CRI-W11-10 and CRI-W70-9 T_1_ plants. TA cloning and sequencing analysis of targeted DNA demonstrated that, the T_1_ plants with CRISPR/Cas9 components, *BnaC.WRKY11.a* and *BnaC.WRKY70.b* were mutated in all of the examined plants, except for *BnaA.WRKY70.a* that showed editing in 7 of 8 plants. We further found that the CRISPR/Cas9 component was crossed out in CRI-W70-10-2 and CRI-W70-3 plants. In CRI-W70-10-2 plants, *BnaA.WRKY70.b* and *BnaC.WRKY70.b* were heterozygous, and both copies showed a “C” insertion in one of the alleles, while *BnaA.WRKY70.a* showed biallelic mutation; Similarly, in CRI-W70-10-3 plants, *BnaA.WRKY70.b* and *BnaA.WRKY70.a* were heterozygous and showed a “C” and “T” insertion in one of the alleles respectively, while *BnaC.WRKY70.b* showed biallelic mutation type with a “C” insertion and combined mutation (2 bp insertion and 3 bp deletion). These results suggested that the genetic mutations in T_0_ plants could be inherited to next generation.

### 2.6. BnWRKY70 Mutants Enhance Resistance to S. sclerotiorum

**Figure 5:**
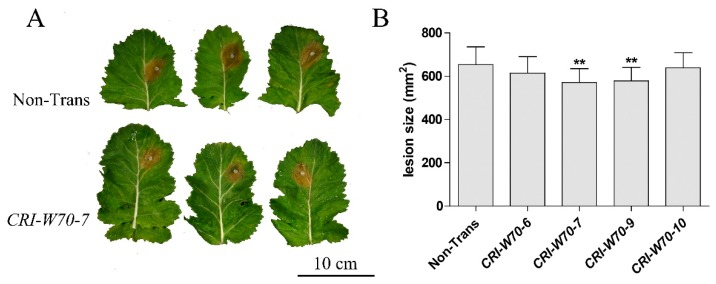
Lesion area on leaves of *BnWRKY70* knockout *B*. *napus* lines inoculated with *S*. *sclerotiorum*. (**A**) Representatives of disease symptom on the Non-Transgenic (Non-Trans), *BnWRKY70* knockout lines. Leaves of 6-week-old plants were inoculated with *S*. *sclerotiorum*. Photos were taken 48 h post-inoculation. (**B**) Lesion area on leaves of *BnWRKY70* overexpression lines. ** indicate that the means are statistically different (*p* < 0.01).

To evaluate the *Sclerotinia* resistance of transgenic plants, *S*. *sclerotiorum* infection was performed on detached leaves of CRI-W70 T_1_ generation plants. The T_1_ plants that mutated three copies of *BnWRKY70* (Table 2) were chose for *Sclerotinia* resistance assessment. Lesion area was measured at 48 hpi. The results showed that, compared with the non-transgenic lines, the lesion areas on the detached leaves of CRI-W70-7 and CRI-W70-9 plants were significantly decreased (Figure 5A,B).

**Figure 6:**
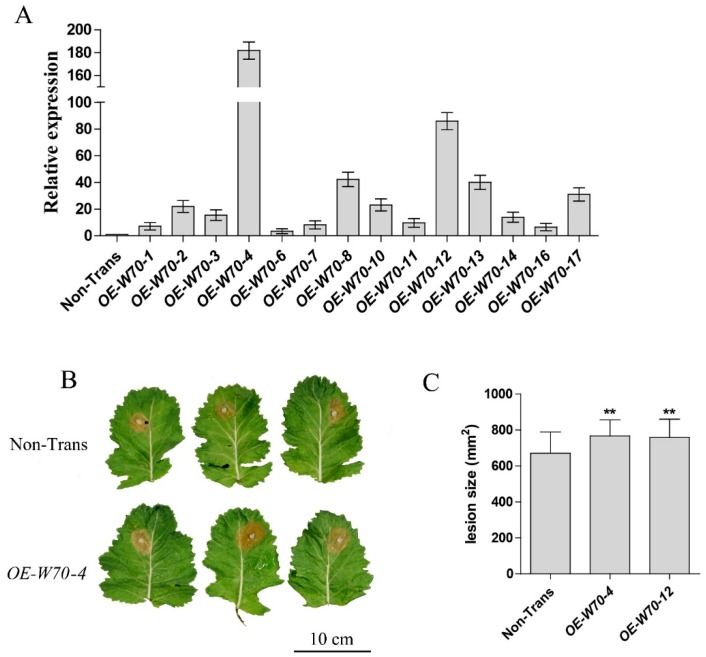
Expression analysis and lesion area on leaves of *BnWRKY70* overexpression *B*. *napus* lines inoculated with *S*. *sclerotiorum.* (**A**) RT-qPCR analysis of *BnWRKY70* expression in overexpression plants. *BnActin7* was used as reference gene. The data shown are the mean of three independent experiments ± standard error (SE). (**B**) Representatives of disease symptom on the Non-Transgenic (Non-Trans), *BnWRKY70* knockout lines. Leaves of 6-week-old plants were inoculated with *S*. *sclerotiorum*. Photos were taken 48 h post-inoculation. (**C**) Lesion area on leaves of *BnWRKY70* overexpression lines. ** indicate that the means are statistically different (*p* < 0.01).

To confirm that the expression level of *BnWRKY70* could affect the *Sclerotinia* resistance in *B*. *napus*, *35S:BnWRKY70* overexpression plants were generated and assessed for *Sclerotinia* resistance. We constructed the binary expression vector pMDC83-*BnWRKY70-GFP* and used *Agrobacterium*-mediated genetic transformation to obtain overexpressed plants. The copy *BnaC.WRKY70.b* was cloned and overexpressed. The expression level of *BnaC.WRKY70.b* in overexpression plants (*OE-W70*) was detected by RT-qPCR with specific primers qW70C08-F and qW70C08-R (Table S2) and 14 overexpression lines were obtained (Figure 6A). The most highly expressed lines *OE-W70-4* and *OE-W70-12* were selected for detached leaves inoculation with *S*. *sclerotiorum*, with the results showing that the lesion areas of the two lines were significantly larger than those of the non-transgenic plants (Figure 6B,C). The above results indicate that *BnWRKY70* plays a negative regulatory role in the defense against *S*. *sclerotiorum* in *B*. *napus*. The resistance of CRI-W11 plants to *S*. *sclerotiorum* was also tested and no significant difference in *S*. *sclerotiorum* resistance was found between *BnWRKY11* knockout mutants and non-transgenic plants (Figure S2).

## 3. Discussion

Many researchers have reported that the CRISPR/Cas9 system mediates targeted genome editing in plants [11,29,30,61,62]. The efficiency of mutations varies depending on the species and constructions of Cas9/sgRNA [22,63]. Ma et al. [64] believed that selection of target with GC contents of approximately 50–70% and with minimal or no base pairing with the sgRNA sequence is desirable. The targets we designed followed these guidelines.

In this research, we demonstrated that the CRISPR/Cas9 system can be an effective tool for multiplex genome editing in *B*. *napus*. As an allotetraploid crop, *B*. *napus* carries two or more copies of one gene in most cases. Thus, multiplex genome editing is necessary for gene knockout plants. Here, we designed 6 targets and constructed 2 gene knockout vectors targeting 6 loci of the *BnWRKY11* and *BnWRKY70* genes. Although both of the Cas9/sgRNA constructions we generated introduced genome editing in T_0_ transgenic *B*. *napus* plants, 2 of the sgRNAs were nonfunctional. Both of the sgRNAs were driven by AtU6-1, while other four were driven by AtU6-29 and AtU3b respectively. If not functioning of the sgRNAs was caused by AtU6-1 promoter need to be confirmed by further experiment. Changing the sgRNA promoters to the *B*. *napus* endogenous promoters and prescreening for functional and efficient sgRNAs might be good solutions to this problem [63].

Three or more independent editing events occurred at the WRKY11-Tgt2 target site *BnaC.WRKY11.a* in some CRI-W11 plants. This result indicated that the regenerated plants were chimeras or the Cas9/sgRNA complexes functioned weakly and continuously after the plant was regenerated from callus. Only one allele of *BnaC.WRKY11.a* in CRI-W11-37 showed targeted genomic deletion even though many mutagenesis occurred at the WRKY11-Tgt2 and WRKY11-Tgt3 target sites simultaneously. Eight *BnWRKY11* transformants with both loci mutated were generated and two *BnWRKY70* transformants with two loci (*BnaA.WRKY11.a* and *BnaA.WRKY11.b*) mutated were obtained in T_0_ plants. This probably because the number of transgenic plants we obtained was insufficient. Nonetheless, mutagenesis might be induced during the growth of plants and all-knockout plants could be generated by selfing or hybridization of transgenic plants for the T_1_ generation. Considering the existence of nonfunctional sgRNA, multiple sgRNAs designed to a given gene are highly recommended for successful editing of targeted genes.

Theoretically, the CRISPR/Cas9 system should continuously function as it exists in a cell until the WT alleles undergo mutation. In our research, we found that the number of editing events induced by the CRISPR/Cas9 system was lower in T_0_ transgenic plants than in T_1_ plants. This result is in accordance with the inference, considering the continuous functioning of CRISPR/Cas9 component, the T_0_ plants should have been developed into complicated chimeras at adult stage. But, when the DNA was sampled from leaves at seedling stage, the editing events detected in T_0_ plants do not complete the genotype of the chimeric plants. Hence, this can explain the detection of new editing events in CRI-W70-10-2 and CRI-W70-10-3 plants, which the CRISPR/Cas9 component were crossed out, showing that the mutations might have been inherited from CRI-W70-10 plants. In addition, the frequent appearance of chimeras in T_1_ plants indicated that most of the mutations occurred after the seed development.

For the transgenic plants with unedited targeted homoeolog(s), screening for plants containing CRISPR/Cas9 component during breeding for continuous editing could be a feasible approach.

Extensive evidence has shown that suppression of the expression of specific genes through RNAi silencing or T-DNA insertion alters the sensitivity to pathogens in plants [49,51,65,66]. Therefore, changing the expression levels of genes could be an effective means to study their functions in disease resistance or for breeding new disease-resistant varieties. Previous studies have found that *WRKY70* is involved in the regulation of leaf senescence [67,68] and BR signaling processes [69] and can participate in plant immune processes by regulating important members of the JA and SA signaling pathways in the plant defense response in *Arabidopsis* [50,70,71,72]. In the present study, except for *BnaC.WRKY70.a*, the other three copies of *BnWRKY70* were mutated in the T_1_ plants of CRI-W70 that we tested. Although homozygous *BnWRKY70* knockout mutants were not obtained in T_1_ generation, mutations of each copy were either homozygous or biallelic for those plants that contain Cas9/sgRNA component, even though in some samples the mutations were chimeric. *S*. *sclerotiorum* infection tests demonstrated that the *BnWRKY70* mutants increased resistance to *S*. *sclerotiorum*. To confirm the negative effects of *BnWRKY70* in *S*. *sclerotiorum* resistance, we constructed *BnWRKY70* overexpression plants. Infection test demonstrated that *BnWRKY70*-overexpressing plants showed a more sensitive phenotype, indicating that the *BnWRKY70* gene may play a negative regulatory role in the response to *S*. *sclerotiorum* in *B*. *napus*. The molecular mechanism of how the *BnWRKY70* gene participates in the disease resistance of rapeseed remains to be further studied.

Because off-targeting has rarely been reported in plants [30,63,73,74], off-target effects were not studied in this study. The risk of off-targeting in transgenic plants that are generated by *Agrobacterium*-mediated transformation could be much lower than in animal cells because the copies of imported foreign genes are fewer in plant cells. Moreover, the targets we designed were highly conserved (data not shown) in the seed sequences [5]. Beyond that, unwanted off-target mutations in plants could be eliminated by crossing the mutant plants with their parental lines [64].

In summary, we demonstrated that the CRISPR/Cas9 system is an effective tool for multiple genome editing in *B*. *napus*. The efficiencies of different sgRNA-induced mutations vary greatly and the mutation types and frequencies induced by CRISPR/Cas9 in *B*. *napus* are similar to those in *Arabidopsis* and rice. Targeted editing of the pathogenic gene can change the defense response in *B*. *napus* to pathogens. Therefore, the CRISPR/Cas9 system is useful for both basic research and disease resistance breeding in *B*. *napus*.

## 4. Materials and Methods

### 4.1. Target Design and Vector Construction for Targeted Gene Mutation

The CRISPR/Cas9-related vectors we used in this research included a CRISPR/Cas9 binary vector and several sgRNA vectors provided by Yaoguang Liu (South China Agricultural University, Guangzhou). The target sequences used to generate sgRNA expression cassettes were selected with the assistance of an online tool called the Optimized CRISPR Plant Design Tool (http://cbi.hzau.edu.cn/cgi-bin/CRISPR) [75] and by referring to common rules [7,75,76]. sgRNA folding was predicted with RNA Folding Form (version 2.3, Energies) [77].

The minimum amount of base pairing formed between the target sequence and sgRNA scaffold or the target sequence itself was selected for genome editing. When the selected target sequences started with the nucleotides “C” or “T”, an extra “A” or “G” nucleotide was added at the 5′ end of the target sequence. To test whether multiple targeted editing can be induced simultaneously by the CRISPR/Cas9 system in transgenic *B*. *napus* plants, we created 2 and 3 gRNA expression cassettes targeting the exon of different copies of *BnWRKY11* and *BnWRKY70*, respectively. In each copy of *BnWRKY11* and *BnWRKY70*, we selected one or two targeting site(s) and designed sgRNAs to target these sites (listed in Table S1). All the target sequences were located in the exon of the open reading frame [78], except for WRKY11-Tgt3, which was located across an exon and an intron.

For mutant identification, we designed one primer pair to amplify a specific locus in most cases or two loci if the identities of two sequences are too similar to distinguish and if the sequences before the target sites share the same length. The construction of CRISPR/Cas9 vectors containing *Cas9* and multiple sgRNA expression cassettes followed the procedure described previously [64]. Briefly, double-stranded target sequences were introduced to the sgRNA expression cassettes by overlapping PCR. Then, the purified PCR products were integrated into pLYCRISPR/Cas9P_35S_-N by a Golden Gate clone [79]. The Cas9/sgRNA constructions were directly used to transform *E*. *coli* competent cells. The positive colonies were selected for sequence identification. The expression of sgRNAs was driven by the AtU3 and/or AtU6 promoter. The ORF of the *Cas9* gene was Gramineae codon optimized and driven by the cauliflower mosaic virus 35S promoter (P_35S_). The CRISPR/Cas9 constructs were introduced to the *Agrobacterium tumefaciens* strain GV3101 through the freezing and thawing method.

### 4.2. Genetic Transformation of B. napus

*B*. *napus* line “J9712” was used as the receptor, which was kindly provided by Professor Yongming Zhou (National Key Laboratory of Crop Genetic Improvement, Huazhong Agricultural University). Transformation of *B*. *napus* was performed as described by De Block et al. [80] with some modification. Briefly, certified, uniform, healthy seeds were surface sterilized with a sodium hypochlorite solution and subsequently rinsed in sterile distilled water. The seeds were germinated on 1/2 MS basal medium with 2% sucrose in darkness. The seedlings were grown at 25 °C in the dark for seven days. Afterward, the hypocotyl (~15 mm) was cut and the explants were made to float in an infection medium [MS medium supplemented with 3% sucrose and 100 μM acetosyringone (AS); pH 5.8] for 20 min. Then, the explants were transferred to a co-cultivation medium (MS medium supplemented with 3% sucrose, 1 mg/L of 2,4-D, 0.3 mg/L of kinetin, 100 μM of AS, 5 mg/L of AgNO_3_ and 8 g/L of agar; pH 5.8) for 3 days. Subsequently, the explants were transferred to a callus induction medium [MS medium supplemented with 3% sucrose, 1 mg/L of 2,4-D, 0.3 mg/L of kinetin, 5 mg/L of AgNO_3_, 500 mg/L of cefotaxime (Cef), 25 mg/L of G418 and 8 g/L of agar; pH 5.8] and incubated at 25 °C. The explants were then transferred to a shoot differentiation medium (MS medium supplemented with 1% glucose, 100 μM of AgNO_3_, 2.0 mg/L of zeatin, 0.1 mg/L of IAA, 500 mg/L of Cef, 25 mg/L of G418 and 8 g/L of agar; pH 5.8) until shoots initialized. Finally, healthy green shoots were transferred to bottles containing a root initiation medium (MS medium supplemented with 1% sucrose and 8 g/L of agar; pH 5.8). Plantlet acclimatization and establishment were performed. The *BnWRKY70* gene BnaC08g27340D (*BnaC.WRKY70.b*) was cloned for overexpression and P_35S_:BnWRKY70-GFP was constructed to generate *BnWRKY70* overexpression plants. The binary expression vector pMDC83 (see vector map on Figure S3) was used in this research.

### 4.3. Mutation Analysis

Genomic DNA was extracted from the transgenic *B*. *napus* plants and wild-type plants using the hexadecyl trimethyl ammonium bromide (CTAB) method [81]. We designed the PCR primers in the flanking region of the Cas9/sgRNA targets and analyzed the targeted mutagenesis by PCR amplification and Sanger sequencing. PCR was performed using Phanta Max Super-Fidelity DNA Polymerase (Vazyme, Nanjing, China). For the regenerated plants, the presence of CRISPR/Cas9 constructs was investigated by PCR with *Cas9* gene primers. For the transformed *B*. *napus* plants, the DNA fragments spanning the Cas9/gRNA target sequences were amplified by PCR and the products were analyzed by TA cloning and sequencing. The primers used for PCR amplification are listed in Table S2.

### 4.4. S. sclerotiorum Infection Assay

*B*. *napus* plants were grown in a field of the experimental farm of Yangzhou University, Jiangsu, China. The *S*. *sclerotiorum* (Lib.) de Bary isolate SS-1 was maintained and cultured on potato dextrose agar (PDA) medium [82]. The uniform agar disk with fungal hyphae was placed on detached leaf surface of 6-week-old *B*. *napus* plants. During inoculation, leaves were kept in a growth tray with a transparent cover to maintain high humidity. The inoculated leaves were transferred to a growth chamber and the lesion sizes were measured at 48-h post-inoculation as descripted in Wu et al. [82].

## Figures and Tables

**Table 1:** The targets and primers designed for *BnWRKY11* and *BnWRKY70* and mutation rates in T_0_ plants.

Target Gene (Number of Transformants)	Copies	Target	Amplification Primer	No. of Plants with Mutations	Mutation Frequency (%)
*BnWRKY11*22	*BnaA.WRKY11.a*	WRKY11-Tgt1, WRKY11-Tgt3	11subF/11subR→11F/11R	0, 7	0, 31.8%
	*BnaC.WRKY11.a*	WRKY11-Tgt2, WRKY11-Tgt3	11F/11R	12, 9	54.5%, 40.9%
*BnWRKY70*8	*BnaC.WRKY70.b*	WRKY70-Tgt1	70F3/70R3	0	0
	*BnaA.WRKY70.b*	WRKY70-Tgt1	70F3/70R3	3	37.5%
	*BnaC.WRKY70.a*	WRKY70-Tgt2	70F1/70R2	0	0
	*BnaA.WRKY70.a*	WRKY70-Tgt3	70F1/70R1	3	37.5%

Tgt, the target sequence used to generate sgRNA expression cassette. The amplify of *BnaA.WRKY11.a* was performed with the primer pair 11subF1/11subR1 first, then subcloned the products with primer pair 11F/11R.

**Table 2:** Sum of the edited T_1_ plants of CRI-W11 and CRI-W70.

Line	Number of Examined Plants	Cas9:sgRNA Constructs ^a^	Number of Edited Plants ^b^			
			*BnWRKY11*			
			*BnaA.WRKY11.a* (WRKY11-Tgt1)	*BnaA.WRKY11.a* (WRKY11-Tgt3)	*BnaC.WRKY11.a* (WRKY11-Tgt2)	*BnaC.WRKY11.a* (WRKY11-Tgt3)
CRI-W11-6	10	10	10	10	0	10
CRI-W11-10	10	10	10	10	0	10
			*BnWRKY70*			
			*BnaA.WRKY70.b*	*BnaA.WRKY70.a*	*BnaC.WRKY70.a*	*BnaC.WRKY70.b*
CRI-W70-6	10	8	8	8	0	8
CRI-W70-7	10	8	8	8	0	8
CRI-W70-9	10	10	10	10	0	10
CRI-W70-10	10	8	10	10	0	10

Tgt means the target sequence used to generate sgRNA expression cassette. ^a^ Cas9:sgRNA construct in the plants was identified by PCR, with the primer pair: Cas9-F/Cas9-R; ^b^ Detailed mutation types for every plant were listed on Table S3.

## Supplementary Materials

The supplementary materials are available online at http://www.mdpi.com/1422-0067/19/9/2716/s1.

## Abbreviations

CRISPR	Clustered, regularly interspaced, palindromic repeat
sgRNA	Single guide RNA
PAM	Protospacer adjacent motif
ZFN	Zinc finger nucleases
TALEN	Transcription activator-like effector nucleases
TFs	Transcription factors
hpi	hours post-inoculation
AS	Acetosyringone
Cef	Cefotaxime
CTAB	Hexadecyl trimethyl ammonium brom
