# Supplementary material for: CRISPR/Cas9-Mediated Multiplex Genome Editing of the BnWRKY11 and BnWRKY70 Genes in Brassica napus L
Source: Int J Mol Sci. 2018 Sep 11;19(9):2716. doi: 10.3390/ijms19092716 (PMC6163266; doi:10.3390/ijms19092716)
Supplement: Supplementary file 1 [file ijms-19-02716-s001.docx]

**Supplementary Materials**

**Table S1.** Summary of the target sequences and the promoters used to drive the expression of sgRNA

| Target Gene | Target | Target sequence (5’-3’) | Promoter |
| --- | --- | --- | --- |
| *BnaA.WRKY11.a* | TgtW11-1F | attGTTTATGAGGAGACGCGGCG | AtU6-1 |
|  | TgtW11-1R | aaacCGCCGCGTCTCCTCATAAA |  |
| *BnaC.WRKY11.a* | TgtW11-2F | gtcACGTTGTTGTTACGCTCGGGG | AtU3b |
|  | TgtW11-2R | aaacCCCCGAGCGTAACAACAACG |  |
| *BnaA.WRKY11.a*  *BnaC.WRKY11.a* | TgtW11-3F | gtcACAGCTCCAAGAGCTCCGAGA | AtU3b |
|  | TgtW11-3R | aaacTCTCGGAGCTCTTGGAGCTG |  |
| *BnaA.WRKY70.b*  *BnaC.WRKY70.b* | TgtW70-1F | gtcACCAACTCGTTCAAGGCCGCG | AtU3b |
|  | TgtW70-1R | aaacCGCGGCCTTGAACGAGTTGG |  |
| *BnaC.WRKY70.a* | TgtW70-2F | attGTATCTCGTCACAGCCGCCGA | AtU6-1 |
|  | TgtW70-2R | aaacTCGGCGGCTGTGACGAGATA |  |
| *BnaA.WRKY70.a* | TgtW70-3F | attGCTCTAGTCACAGCCGTCGA | AtU6-29 |
|  | TgtW70-3R | aaacTCGACGGCTGTGACTAGAG |  |

**Table S2.** Primers used for mutagenesis analysis and RT-qPCR analysis in this paper

| Primer | Sequence |
| --- | --- |
| 11F | AGACCAACCAATCCATCATGGC |
| 11R | TCGTGCTCTRCGCATCTYC |
| 11subF | AGACCAACCAATCCATCATG |
| 11subR | CTAACTAATAATCCCGCGGTAATAG |
| 70F1 | AGCCCATACCAAGAGAGGAAAACAT |
| 70R1 | TCAACTAATATGTAAAGGGATTAAGC |
| 70R2 | CACTGATTGATTTAAGTCAGAGGCTGT |
| 70F3 | CATATCACCCACACCAATAGAGAG |
| 70R3 | GAGGAGGAGGAGACGGGTTCTAAGG |
| 70subR3 | ATCCCGGCAACATGTCACATATCA |
| qActin-F | TCTTCCTCACGCTATCCTCCG |
| qActin-R | AGCCGTCTCCAGCTCTTGC |
| qW70C08-F | ACCACGATGAAGGAAGAGGAAGAG |
| qW70C08-R | ACCACACCCATAAATAGCGACATGA |

**Table S3.** Mutagenesis of *B*. *napus* genome in T_1_ plants

| Gene | Sequences | plants |
| --- | --- | --- |
| *BnaA.WRKY11.a* | ggttcc**CCC**CGC-CGCGTCTCCTCATAAACacca//--140 bp--//gatcCAGCTCCAAGAGCTCCG-AGA**TGG**acttc  ggttcc**CCC**CGC-CGCGTCTCCTCATAAACacca//--140 bp--//gatcCAGCTCCAAGAGCTCCGAAGA**TGG**acttc \, +1  ggttcc**CCC**CGC-CGCGTCTCCTCATAAACacca//--140 bp--//gatcCAGCTCCAAGAGCTCCGTAGA**TGG**acttc \, +1  ggttcc**CCC**CGC-CGCGTCTCCTCATAAACacca//--140 bp--//gatcCAGCTCCAAGAGCTCCGCAGA**TGG**acttc \, +1  ggttcc**CCC**CGC-CGCGTCTCCTCATAAACacca//--140 bp--//gatcCAGCTCCAAGAGCTCCGAAGA**TGG**acttc \, +1  ggttcc**CCC**CGC-CGCGTCTCCTCATAAACacca//--140 bp--//gatcCAGCTCCAAGAGCTCCGTAGA**TGG**acttc \, +1  ggttcc**CCC**CGC-CGCGTCTCCTCATAAACacca//--140 bp--//gatcCAGCTCCAAGAGCTCCGAAGA**TGG**acttc \, +1  ggttcc**CCC**CGC-CGCGTCTCCTCATAAACacca//--140 bp--//gatcCAGCTCCAAGAGCTCCGTAGA**TGG**acttc \, +1  ggttcc**CCC**CGC-CGCGTCTCCTCATAAACacca//--140 bp--//gatcCAGCTCCAAGAGCTCCGCAGA**TGG**acttc \, +1  ggttcc**CCC**CGC-CGCGTCTCCTCATAAACacca//--140 bp--//gatcCAGCTCCAAGAGCTCCGAAGA**TGG**acttc \, +1  ggttcc**CCC**CGC-CGCGTCTCCTCATAAACacca//--140 bp--//gatcCAGCTCCAAGAGCTCCGTAGA**TGG**acttc \, +1  ggttcc**CCC**CGC-CGCGTCTCCTCATAAACacca//--140 bp--//gatcCAGCTCCAAGAGCTCC--AGA**TGG**acttc \, -1  ggttcc**CCC**CGC-CGCGTCTCCTCATAAACacca//--140 bp--//gatcCAGCTCCAAGAGCTCCGAAGA**TGG**acttc \, +1  ggttcc**CCC**CGC-CGCGTCTCCTCATAAACacca//--140 bp--//gatcCAGCTCCAAGAGCTCCGCAGA**TGG**acttc \, +1  ggttcc**CCC**CGC-CGCGTCTCCTCATAAACacca//--140 bp--//gatcCAGCTCCAAGAGCTCCGAAGA**TGG**acttc \, +1  ggttcc**CCC**CGC-CGCGTCTCCTCATAAACacca//--140 bp--//gatcCAGCTCCAAGAGCTCCGGAGA**TGG**acttc \, +1  ggttcc**CCC**CGC-CGCGTCTCCTCATAAACacca//--140 bp--//gatcCAGCTCCAAGAGCTCCGCAGA**TGG**acttc \, +1  ggttcc**CCC**CGC-CGCGTCTCCTCATAAACacca//--140 bp--//gatcCAGCTCCAAGAGCTCCGTAGA**TGG**acttc \, +1  ggttcc**CCC**CGC-CGCGTCTCCTCATAAACacca//--140 bp--//gatcCAGCTCCAAGAGCTCCGAAGA**TGG**acttc \, +1 | WT allele(Tgt1/Tgt3)  CRI-W11-6-1  CRI-W11-6-2  CRI-W11-6-3  CRI-W11-6-4  CRI-W11-10-1  CRI-W11-10-2  CRI-W11-10-3  CRI-W11-10-4 |
| *BnaC.WRKY11.a* | actaa**CCC**CCC----CGAGCGTAACAACAACGttga//--260 bp--//gatcCAGCTCCAAGAGCTCCG-AGA**TGG**acttc  actaa**CCC**CCCA---CGAGCGTAACAACAACGttga//--260 bp--//gatcCAGCTCCAAGAGCTCCGGAGA**TGG**acttc +1, +1  actaa**CCC**CCCT---CGAGCGTAACAACAACGttga//--260 bp--//gatcCAGCTCCAAGAGCTCCGAAGA**TGG**acttc +1, +1  actaa**CCC**CC-----CGAGCGTAACAACAACGttga//--260 bp--//gatcCAGCTCCAAGAGCTCCGAAGA**TGG**acttc -1, +1  actaa**CCC**CCCAAC-CGAGCGTAACAACAACGttga//--260 bp--//gatcCAGCTCCAAGAGCTCCGAAGA**TGG**acttc +3, +1  actaa**CCC**CCCT---CGAGCGTAACAACAACGttga//--260 bp--//gatcCAGCTCCAAGAGCTCCGCAGA**TGG**acttc +1, +1  actaa**CCC**CCCA---CGAGCGTAACAACAACGttga//--260 bp--//gatcCAGCTCCAAGAGCTCCG-AGA**TGG**acttc +1, \  actaa**CCC**CCCT---CGAGCGTAACAACAACGttga//--260 bp--//gatcCAGCTCCAAGAGCTCCGCAGA**TGG**acttc +1, +1  actaa**CCC**CCCA---CGAGCGTAACAACAACGttga//--260 bp--//gatcCAGCTCCAAGAGCTCCGGAGA**TGG**acttc +1, +1  actaa**CCC**CCCA----GAGCGTAACAACAACGttga//--260 bp--//gatcCAGCTCCAAGAGCTCCG-AGA**TGG**acttc +1-1, \ | WT allele (Tgt2/Tgt3)  CRI-W11-6-1  CRI-W11-6-2  CRI-W11-6-3  CRI-W11-6-4  CRI-W11-10-1  CRI-W11-10-2  CRI-W11-10-3  CRI-W11-10-4 |
| *BnaA.WRKY70.b* | caaagcaataaagctaaaagctaggcaCCAACTCGTTCAAGGCC-GCG**AGG**tggccactaagcttcagcatctcctctttcaac  caaagcaataaagctaaaagctaggcaCCAACTCGTTCAAGGCCCGCG**AGG**tggccactaagcttcagcatctcctctttcaac +1  caaagcaataaagctaaaagctaggcaCCAACTCGTT--------GCG**AGG**tggccactaagcttcagcatctcctctttcaac -7  caaagcaataaagctaaaagctaggcaCCAACTCGTTCAAGGC--GCG**AGG**tggccactaagcttcagcatctcctctttcaac -1  caaagcaataaagctaaaagctaggcaCCAACTCGTTCAAGGCCCGCG**AGG**tggccactaagcttcagcatctcctctttcaac +1  caaagcaataaagctaaaagctaggcaCCAACTCGTTCAAGGCCCGCG**AGG**tggccactaagcttcagcatctcctctttcaac +1  caaagcaataaagctaaaagctaggcaCCAACTCGTTCAAGGCCCGCG**AGG**tggccactaagcttcagcatctcctctttcaac +1  caaagcaataaagctaaaagctaggcaCCAACTCGTTCAAGGCCAGCG**AGG**tggccactaagcttcagcatctcctctttcaac +1  caaagcaataaagctaaaagctaggcaCCAACTCGTTCAAGGCCCGCG**AGG**tggccactaagcttcagcatctcctctttcaac +1  caaagcaataaagctaaaagctaggcaCCAACTCGTTCAAGGCCCGCG**AGG**tggccactaagcttcagcatctcctctttcaac +1  caaagcaataaagctaaaagctaggcaCCAACTCGTTCAAGGCCCGCG**AGG**tggccactaagcttcagcatctcctctttcaac +1  caaagcaataaagctaaaagctaggcaCCAACTCGTTCAAG----GCG**AGG**tggccactaagcttcagcatctcctctttcaac -3  caaagcaataaagctaaaagctaggcaCCAACTCGTTCAAGGCCCGCG**AGG**tggccactaagcttcagcatctcctctttcaac +1  caaagcaataaagctaaaagctaggcaCCAACTCGTTCAAGGCCTTCCCCACTGCTGCCTCCCGTAGGAGTCTGGGCCGTGTCTCAGTCCCAGTGTGGCTGATCAACGCG**AGG**tggccactaagcttcagcatctcctctttcaac +64  caaagcaataaagctaaaagctaggcaCCAACTCGTTCAAGGCCCGCG**AGG**tggccactaagcttcagcatctcctctttcaac +1  caaagcaataaagctaaaagctaggcaCCAACTCGTTCAAGGCCCGCG**AGG**tggccactaagcttcagcatctcctctttcaac +1 | WT allele (Tgt1)  CRI-W70-6-1  CRI-W70-6-2  CRI-W70-6-3  CRI-W70-7-1  CRI-W70-7-2  CRI-W70-7-3  CRI-W70-9-1  CRI-W70-9-2  CRI-W70-9-3  CRI-W70-10-1  CRI-W70-10-2  CRI-W70-10-3 |
| *BnaA.WRKY70.a* | tgattccttcgaacccatctccccgtctGCTCTAGTCACAGCCGT-CGA**AGG**ctctcaaaatgcttcctgcgacaacgacggca  tgattccttcgaacccatctccccgtctGCTCTAGTCACAGCCGTTCGA**AGG**ctctcaaaatgcttcctgcgacaacgacggca +1  tgattccttcgaacccatctccccgtctGCTCTAGTCACAGCCGTTCGA**AGG**ctctcaaaatgcttcctgcgacaacgacggca +1  tgattccttcgaacccatctccccgtctGCTCTAGTCACAGCCGTTCGA**AGG**ctctcaaaatgcttcctgcgacaacgacggca +1  tgattccttcgaacccatctccccgtctGCTCTAGTCACAGCCGTGCGA**AGG**ctctcaaaatgcttcctgcgacaacgacggca +1  tgattccttcgaacccatctccccgtctGCTCTAGTCACAGCCGTTCGA**AGG**ctctcaaaatgcttcctgcgacaacgacggca +1  tgattccttcgaacccatctccccgtctGCTCTAGTCACAGCCGTTCGA**AGG**ctctcaaaatgcttcctgcgacaacgacggca +1  tgattccttcgaacccatctccccgtctGCTCTAGTCACAGCCGTTCGA**AGG**ctctcaaaatgcttcctgcgacaacgacggca +1  tgattccttcgaacccatctccccgtctGCTCTAGTCACAGCCGTTCGA**AGG**ctctcaaaatgcttcctgcgacaacgacggca +1  tgattccttcgaacccatctccccgtctGCTCTAGTCACAGCCGTGCGA**AGG**ctctcaaaatgcttcctgcgacaacgacggca +1  tgattccttcgaacccatctccccgtctGCTCTAGTCACAGCCGTGCGA**AGG**ctctcaaaatgcttcctgcgacaacgacggca +1  tgattccttcgaacccatctccccgtctGCTCTAGTCACAGCCGTTCGA**AGG**ctctcaaaatgcttcctgcgacaacgacggca +1  tgattccttcgaacccatctccccgtctGCTCTAGTCACA------CGA**AGG**ctctcaaaatgcttcctgcgacaacgacggca -5  tgattccttcgaacccatctccccgtctGCTCTAGTCA----------A**AGG**ctctcaaaatgcttcctgcgacaacgacggca -9  tgattccttcgaacccatctccccgtctGCTCTAGTCACAGCCGTGCGA**AGG**ctctcaaaatgcttcctgcgacaacgacggca +1  tgattccttcgaacccatctccccgtctGCTCTAGTCACAGCCGTTCGA**AGG**ctctcaaaatgcttcctgcgacaacgacggca +1  tgattccttcgaacccatctccccgtctGCTCTAGTCACAGCCGTACGA**AGG**ctctcaaaatgcttcctgcgacaacgacggca +1  tgattccttcgaacccatctccccgtctGCTCTAGTCACAGCCGTTCGA**AGG**ctctcaaaatgcttcctgcgacaacgacggca +1  tgattccttcgaacccatctccccgtctGCTCTAGTCACAGC----CGA**AGG**ctctcaaaatgcttcctgcgacaacgacggca -3  tgattccttcgaacccatctccccgtctGCTCTAGTCACAGCCGTTCGA**AGG**ctctcaaaatgcttcctgcgacaacgacggca +1 | WT allele (Tgt3)  CRI-W70-6-1  CRI-W70-6-2  CRI-W70-6-3  CRI-W70-7-1  CRI-W70-7-2  CRI-W70-7-3  CRI-W70-9-1  CRI-W70-9-2  CRI-W70-9-3  CRI-W70-10-1  CRI-W70-10-2  CRI-W70-10-3 |
| *BnaC.WRKY70.b* | acaaagcaataacgctaaaagctagggaCCAACTCGTTCAAGGCC-GCG**AGG**tggccactgagcttcagcaactcctctttcaa  acaaagcaataacgctaaaagctagggaCCAACTCGTTCAAGGCCCGCG**AGG**tggccactgagcttcagcaactcctctttcaa +1  acaaagcaataacgctaaaagctagggaCCAACTCGTTCAAGGCCCGCG**AGG**tggccactgagcttcagcaactcctctttcaa +1  acaaagcaataacgctaaaagctagggaCCAACTCGTTCAAGGCCCGCG**AGG**tggccactgagcttcagcaactcctctttcaa +1  acaaagcaataacgctaaaagctagggaCCAACTCGTTCAAGGCC--GT**AGG**tggccactgagcttcagcaactcctctttcaa +2-3  acaaagcaataacgctaaaagctagggaCCAACTCGTTC-------GCG**AGG**tggccactgagcttcagcaactcctctttcaa -6  acaaagcaataacgctaaaagctagggaCCAACTCGTTCAAGGCCAGCG**AGG**tggccactgagcttcagcaactcctctttcaa +1  acaaagcaataacgctaaaagctagggaCCAACTCGTTCAAGGCCCGCG**AGG**tggccactgagcttcagcaactcctctttcaa +1  acaaagcaataacgctaaaagctagggaCCAACTCGTTCAAGGCCCGCG**AGG**tggccactgagcttcagcaactcctctttcaa +1  acaaagcaataacgctaaaagctagggaCCAACTCGTTCAAG----GCG**AGG**tggccactgagcttcagcaactcctctttcaa -3 | WT allele (Tgt1)  CRI-W70-10-1  CRI-W70-10-2  CRI-W70-10-3  CRI-W70-10-4  CRI-W70-10-5 |

Note: The PAM are shown in bold blue letters; red dashes mark the deletions; inserted nucleotide is marked by green letter. The numbers on the right show the type of mutation and how many nucleotides are involved, with “−” and “+” indicating deletion or insertion of the given number of nucleotides, respectively.

**Table S4.** Mutation rates in T_0_ transgenic plants of *BnWRKY11*

| **Target Gene** | **No. of Transgenic Plants** | **Mutation efficiency (%)^a^** | | | |
| --- | --- | --- | --- | --- | --- |
|  |  | *BnaA.WRKY11.a* | | *BnaC.WRKY11.a* | |
|  |  | Tgt1 | Tgt3 | Tgt2 | Tgt3 |
| *BnWRKY11* | *CRI-W11-5* | -^b^ | - | 11.7 | - |
|  | *CRI-W11-6* | - | - | - | - |
|  | *CRI-W11-7* | - | 7.1 | 47.7 | 49.9 |
|  | *CRI-W11-9* | - | - | - | - |
|  | *CRI-W11-10* | - | - | - | - |
|  | *CRI-W11-11* | - | - | - | - |
|  | *CRI-W11-12* | - | - | - | - |
|  | *CRI-W11-13* | - | - | 38.1 | 35.6 |
|  | *CRI-W11-14* | - | - | 9.9 | - |
|  | *CRI-W11-15* | - | 92.7 | 85.3 | 89.2 |
|  | *CRI-W11-17* | - | - | - | - |
|  | *CRI-W11-19* | - | - | 72.1 | 48.5 |
|  | *CRI-W11-20* | - | - | - | - |
|  | *CRI-W11-21* | - | 3.9 | 50.0 | 43.0 |
|  | *CRI-W11-24* | - | - | - | - |
|  | *CRI-W11-25* | - | 63.1 | 82.3 | 59.5 |
|  | *CRI-W11-26* | - | 18.4 | 80.8 | 42.3 |
|  | *CRI-W11-27* | - | 48.1 | 77.1 | 46.8 |
|  | *CRI-W11-29* | - | 72.8 | 90.1 | 75.0 |
|  | *CRI-W11-31* | - | - | - | - |
|  | *CRI-W11-32* | - | - | - | - |
|  | *CRI-W11-37* | - | 41.8 | 22.9^c^ | |

Plant with multiple genome modifications was shown with shade of yellow.

a The mutation efficiency was evaluated by TIDE, with p-value <0.001.

b If the p-value of predicted mutation efficiency given by TIDE was larger than 0.001, we label the efficiency as non-edited sequence with “-“.

c The *BnaC.WRKY11.a* gene of *CRI-W11-37* was mutated with a 302 bp deletion between WRKY11-Tgt2 and WRKY11-Tgt3, so the number was not accurate.

**Table S5.** Mutation rates in T_0_ transgenic plants of *BnWRKY11*

| **Target Gene** | **Transgenic Plants** | **Mutation efficiency (%)^a^** | | |
| --- | --- | --- | --- | --- |
|  |  | *BnaA.WRKY70.b* | | *BnaA.WRKY70.a* |
|  |  | Tgt1 | | Tgt3 |
| *BnWRKY70* | *CRI-W70-6* | 6.0 | - | |
|  | *CRI-W70-7* | - | 19.4 | |
|  | *CRI-W70-9* | - | - | |
|  | *CRI-W70-10* | 59.5 | 38.8 | |
|  | *CRI-W70-12* | 22.6 | 55.4 | |
|  | *CRI-W70-13* | - | - | |
|  | *CRI-W70-18* | - | - | |
|  | *CRI-W70-19* | - | - | |

Plant with multiple genome modifications was shown with shade of yellow.

a The mutation efficiency was evaluated by TIDE, with p-value <0.001.

b If the p-value of predicted mutation efficiency given by TIDE was larger than 0.001, we label the efficiency as non-edited sequence with “-“.


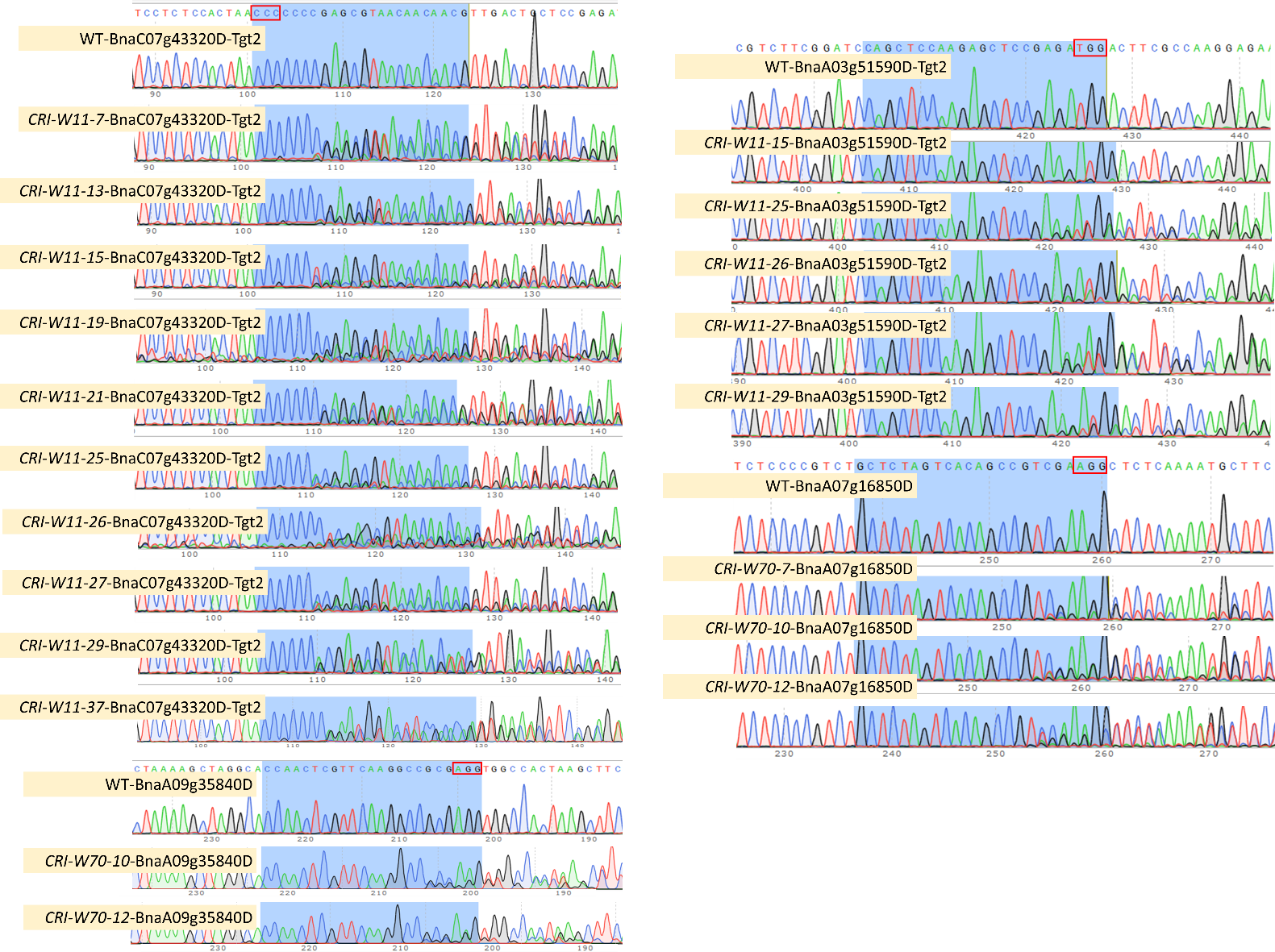


**Figure S1.** Part sequence chromatograms of mutated amplicons

Target sequences were showed with shadow, the PAMs were showed in red boxes.

**Figure S2.** Lesion area on leaves of *BnWRKY11* knockout and overexpression lines inoculated with *Sclerotinia* *sclerotiorum*


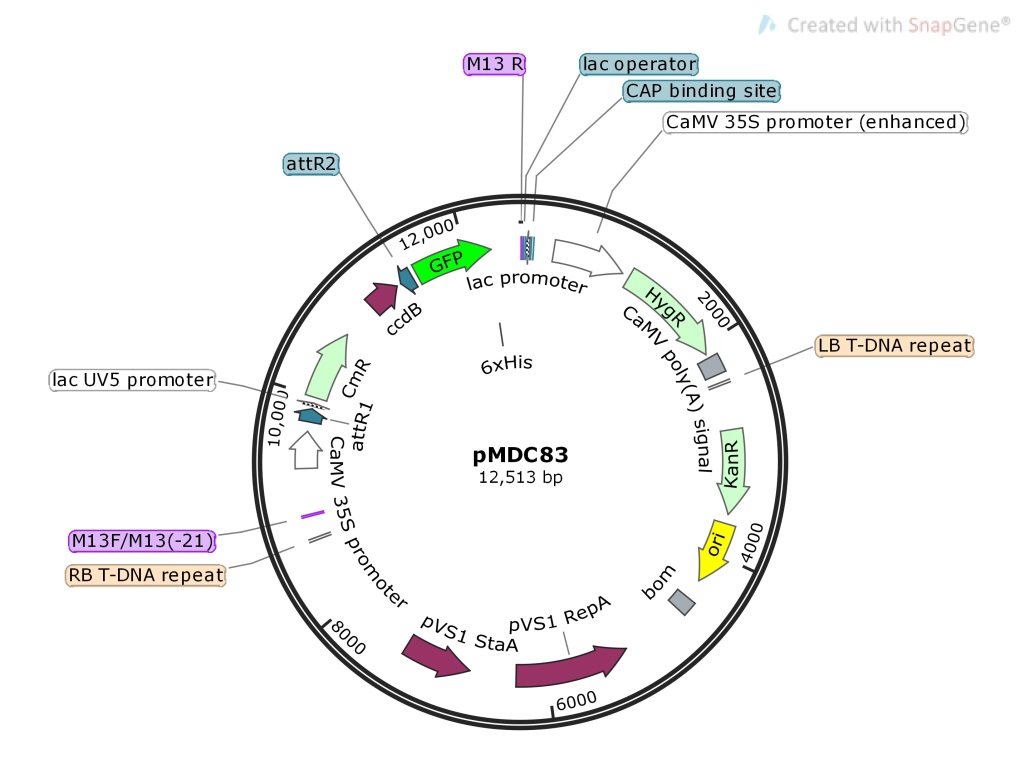


**Figure S3.** Schematic representation of pMDC83
